# Supplementary material for: High-Temperature Quantum Hall Effect in Graphite-Gated Graphene Heterostructure Devices with High Carrier Mobility
Source: Nanomaterials (Basel). 2022 Oct 26;12(21):3777. doi: 10.3390/nano12213777 (PMC9654316; doi:10.3390/nano12213777)
Supplement: Supplementary file 1 [file nanomaterials-12-03777-s001.zip › nanomaterials-1967898-supplementary.pdf]

nanomaterials

Supplementary Materials

High-Temperature Quantum Hall Effect in Graphite-Gated Graphene Heterostructure Devices with High Carrier Mobility

Siyu Zhou †, Mengjian Zhu *,†, Qiang Liu, Yang Xiao, Ziru Cui and Chucai Guo *

College of Advanced Interdisciplinary Studies & Hunan Provincial Key Laboratory of Novel Nano-Optoelectronic Information Materials and Devices, National University of Defense Technology, Changsha 410073, China

* Correspondence: zhumengjian11@nudt.edu.cn (M.Z.); gcc_1981@163.com (C.G.)

† These authors contributed equally to this work.

Citation: Zhou, S.; Zhu, M.; Liu, Q.; Xiao, Y.; Cui, Z.; Guo, C. High-Temperature Quantum Hall Effect in Graphite-Gated Graphene Heterostructure Devices with High Carrier Mobility. *Nanomaterials* **2022**, *12*, 3777. https://doi.org/10.3390/nano12213777

Academic Editor: Jean-Pierre Bucher

Received: 28 September 2022
Accepted: 22 October 2022
Published: 26 October 2022

Publisher's Note: MDPI stays neutral with regard to jurisdictional claims in published maps and institutional affiliations.

Copyright: © 2022 by the authors. Licensee MDPI, Basel, Switzerland. This article is an open access article distributed under the terms and conditions of the Creative Commons Attribution (CC BY) license (https://creativecommons.org/licenses/by/4.0/).

1. Device fabrication

The fabrication process in this experiment uses polycarbonate (PC) film as the colloidal adsorption material during transfer, and polydimethylsiloxane (PDMS) as the support layer on the glass slide [1]. The process of preparing PDMS is as follows: mix the main agent and curing agent according to the weight ratio of 10:1; After that, the prepared solution will be fully stirred, and a large number of bubbles will be generated during the mixing process. Wait for the bubbles to disappear by placing the solution in vacuum box, and then drop the prepared solution on the glass slide and heat it at 125°C for 20 minutes to completely solidify it as showed in Figure S1(a).

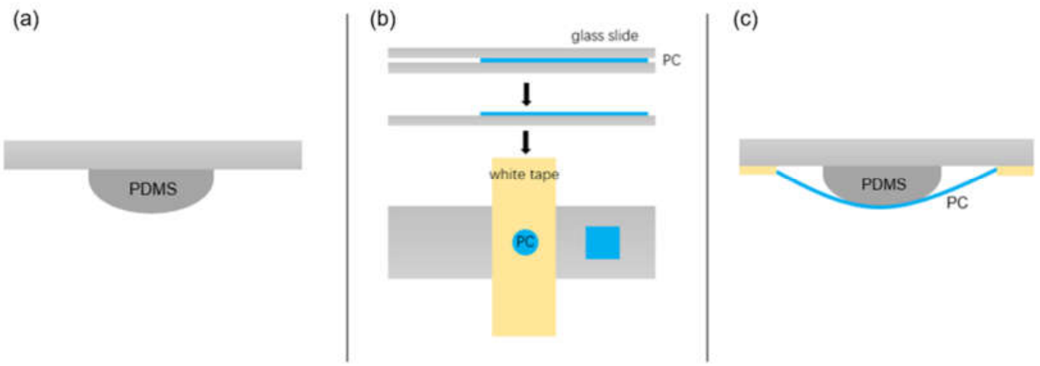

Figure S1. (a-c) Preparation of slide for transfer.

When preparing PC solution, mix PC particles with $CHCl_3$ according to the weight ratio of 6:94, and then fully stir the prepared solution at 75℃ for 24h through magnetic stirrer. After that, drop 2-3 drops of PC solution on the glass slide, squeeze another glass slide to flatten the solution, and then separate the two glass slides in parallel. After standing for a few minutes, PC film can be formed; On the glass slide after film formation, use a blade to draw a square of about 1cm×1cm; First, use white tape to stick away the surrounding film, leaving a square film; Then use the puncher to punch a round hole on the white tape, align it with the square film, and quickly connect it after full fitting. At this time, PC film is obtained. Then align the PC film with the round hole on the tape to PDMS and try to form it at one time to get the desired structure in Figure S1(c).

When fabricating the device, an optical microscope and a motor-driven lifting platform are used, which can carry out the lifting operation with a graduation of 0.001mm. We choose hBN flakes with a thickness of about 30-40nm, place the samples on the stage, heat them to 90℃, and then pick them up with transfer slides. Then we select the monolayer graphene, align it with the hBN on the glass slide and pick it up. Repeat the process to pick up another hBN and graphite, so we can get the hBN/MLG/hBN/FLG heterostructure. Then we select a new silicon oxide wafer, heat it to 180℃, release the hBN/MLG/hBN/FLG on this wafer. After that, we dissolve the residual PC in chloroform ($CHCl_3$) for 2 minutes, wash it with isopropanol (IPA) and dry it with nitrogen.

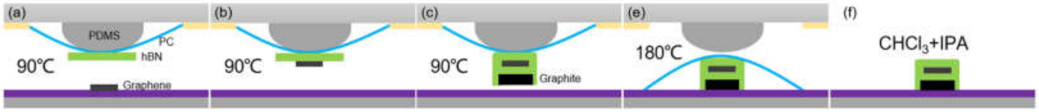

Figure S2. (a-f) Fabrication process of hBN/MLG/hBN/FLG heterostructure.

In order to further remove the residual PC, we also anneal the prepared samples at 300℃ under high temperature and high vacuum for 3 hours. We use EBL technology to expose the required metal electrode shape, and then use CHF_3 (20 sccm) and O_2 (10 sccm) and Ar (10 sccm) induction coupled plasma [2] (RF1: 50 W, RF2: 0 W) to selectively etch hBN to expose the edge of graphene in the middle layer. The metal electrode of 5 nm Cr / 50 nm Au is evaporated on the sample by electron beam evaporation, so as to realize one-dimensional contact between graphene and metal electrode [3]. Then, the shape similar to Hall bar is made in the same way. Finally, the device is placed on the PCB through aluminum wire bonding.

2. Raman spectroscopy

We use Raman spectroscopy to characterize our devices. We use a Witec confocal micro-Raman spectroscopy with a solid-state laser at 532 nm wavelength to test at 10 mW laser power under a 100× objective.

3. Electrical measurements

The electrical measurement set-up is illustrated as below Figure S3. A constant current is passing through the graphene Hall bar, the longitudinal and transverse resistance are measured as R_{xx} and R_{xy}, respectively.

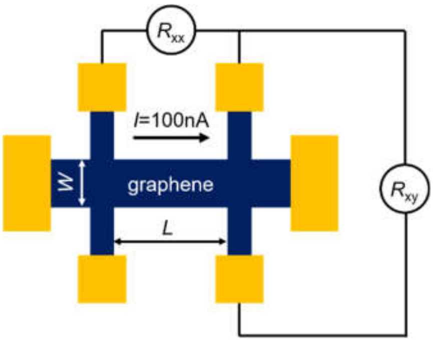

Figure S3. The method of electrical measurement.

The conductivity σ of graphene device is determined by the measured longitudinal resistance R_{xx} using below formula:

$$\sigma = \frac{1}{\rho} = \frac{1}{R_{xx}}\frac{L}{W} = en\mu$$

Where ρ is the resistivity, L and W are the length and width of the device, e is the elementary charge, n is the carrier concentration and μ is the field-effect carrier mobility.

4. Transport measurements of additional graphene Hall bars

In order to compare the device performance of graphene on different substrates, we fabricate two types of graphene Hall bars: (1) Si/300nm-SiO_2/graphene/hBN device and (2) graphite/hBN/graphene/hBN device, as shown in below Figure S4. The thickness of hBN in device #2 is ~30nm. The measured resistivities of two graphene devices as a function of gate voltage are plotted in Figure S4(c) and (d). Considering the capacitance of device #1 is 10 times less than the capacitance of device #2, the x-axis in Figure S4(c) and (d) represent the same carrier concentration range (n=CV_g). One can see that the resistivity peak in the graphite-gated device is much shaper than the SiO_2 supported device, which implies higher carrier mobility and less carrier fluctuation in device #2. The substrate-induced carrier doping in graphene can be determined by the gate voltage corresponding to the Dirac point (n_{DP}=CV_{DP}). For graphene on SiO_2 substrate, the doping level is $1.4x10^{12}cm^{-2}$, which is nearly one order of magnitude larger than graphite-gated device ($1.6x10^{11}cm^{-2}$). Therefore, we can infer that the substrate-induced carrier scattering in graphite-gated graphene device is significantly weaker than the device directly deposited on SiO_2 substrate.

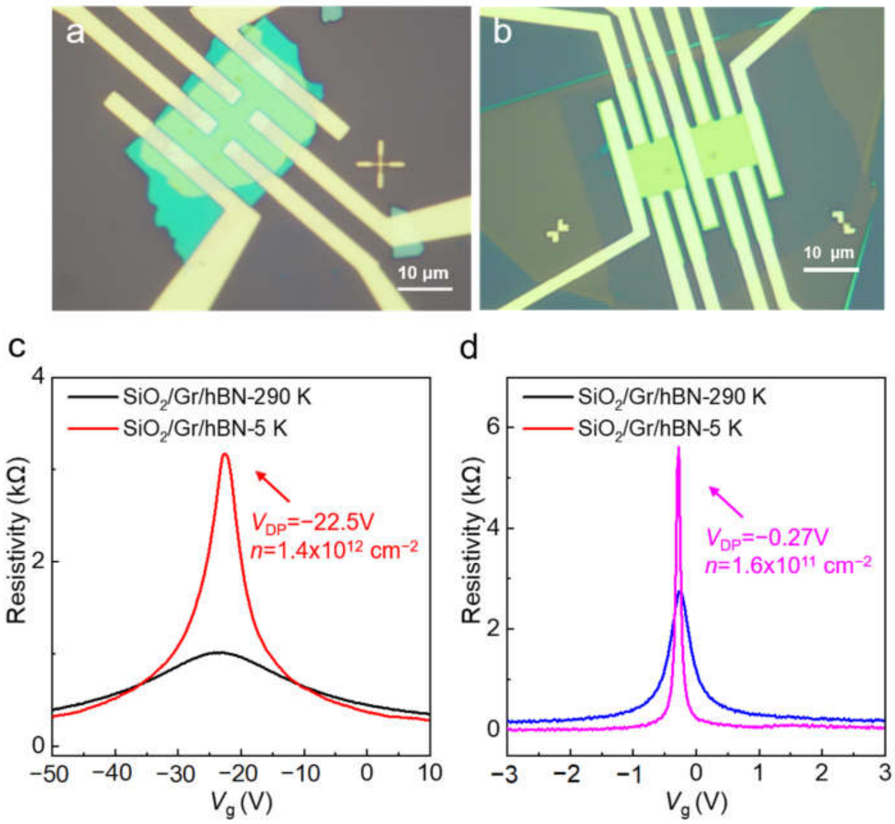

Figure S4. Graphene Hall bar devices on different substrates. Optical microscopy images of **(a)** graphene device on SiO_2 substrate and **(b)** hBN encapsulated graphene (Gr) device on graphite (Gra) local gate. Measured longitudinal resistivity of **(c)** SiO_2-supported graphene device and **(d)** graphite-gated graphene device. V_{DP} refers to the gate voltage for the Dirac point of graphene.

To qualitatively analysis the substrate-induced carrier fluctuation in graphene, we carried out similar analysis of the transport data with Figure 2 in the manuscript, as shown in Figure S5. The accessible carrier fluctuation in SiO_2-supported graphene device is about $3x10^{11}cm^{-2}$ at room temperature and $10^{11}cm^{-2}$ at 5 K. Both of the values are 5 times larger than the carrier fluctuation level in graphite-gated graphene devices, as shown in Figure S5(a-b). As a result of reduced scattering, graphite-gated device exhibit much higher field effect carrier-mobility (~$3x10^4$ $cm^2/V{\cdot}s$ at 290K and ~$2x10^5$ $cm^2/V{\cdot}s$ at 5K for device #2) than that of SiO_2-supported graphene devices (~$9x10^3$ $cm^2/V{\cdot}s$ at 290K and ~$3x10^4$ $cm^2/V{\cdot}s$ at 5K for device #1), as shown in Figure S5(c-d).

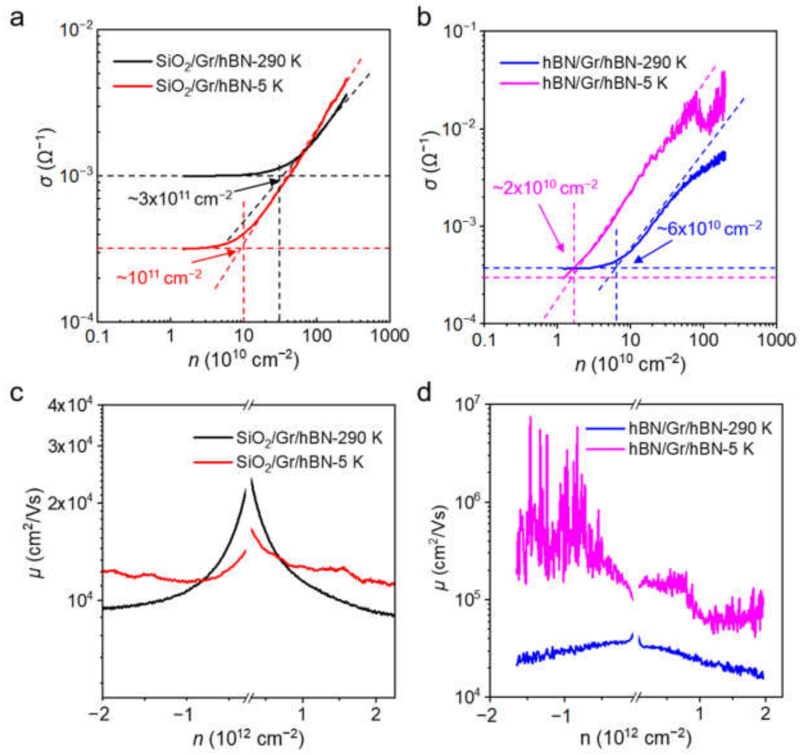

Figure S5. Carrier concentration dependence of transport properties of graphene devices on different substrates. The conductivity as a function of the charge carrier density n for **(a)** SiO_2-supported graphene devices and **(b)** graphite-gated graphene device. The residual charge carrier fluctuation is calculated by the intercept of two lines. Carrier mobility μ as function of charge carrier density **(c)** SiO_2-supported graphene devices and **(d)** graphite-gated graphene device.

5. Transport measurements at T = 200 K and B = 12 T

Higher the temperature goes, the mobility is decreasing as is shown in Figure 2(a) and (d) in the main text. We also measured the R_{xx} and R_{xy} at 200 K under a big magnetic field of 12 T, see Figure S6, we can barely see the quantum hall plateau values of $h/\pm 2e^2$. With the condition of $\mu \cdot B \gg 1$ to observe the hall plateaus, as the mobility decreased, we need to reach a much bigger magnetic field (at least > 12 T) in order to see the hall plateaus.

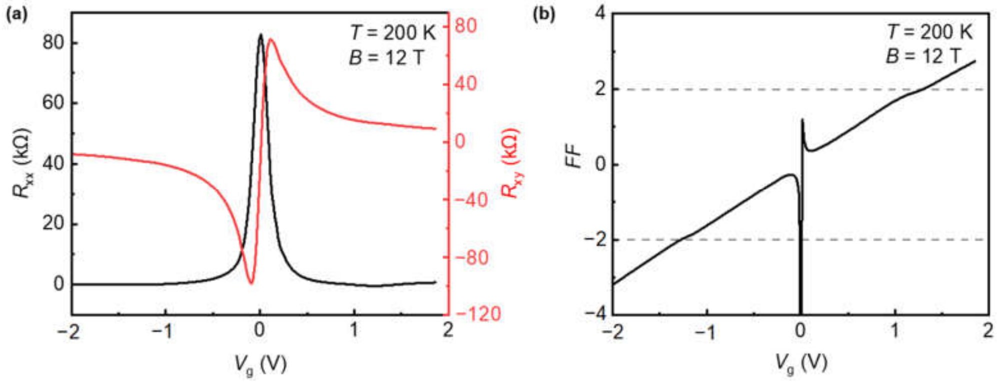

Figure S6. (a) R_{xx} and R_{xy} at 200 K under a big magnetic field of 12 T. **(b)** Filling Factor of R_{xy} as a function of graphite-gate voltage at T = 200 K and B =12 T.

6. Transport measurements with 0.1 T at 100 K and 150 K

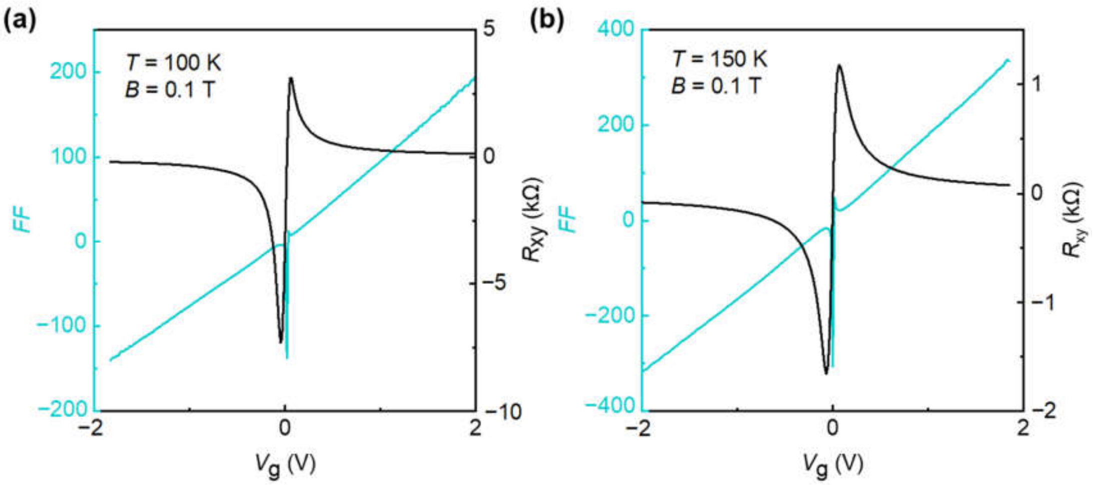

Figure S7. (a) R_{xy} and Filling Factor as a function of graphite-gate voltage at T = 100 K at B = 0.1 T. **(b)** Same as (a) but measured at T = 150 K.

We plot the data with 0.1 T at 100 K and 150 K, we cannot observe the hall plateaus at such high temperature with low magnetic field.

References

1. Kim, K.; Yankowitz, M.; Fallahazad, B.; Kang, S.; Movva, H.C.P.; Huang, S.; Larentis, S.; Corbet, C.M.; Taniguchi, T.; Watanabe, K.; et al. van der Waals Heterostructures with High Accuracy Rotational Alignment. *Nano Lett.* **2016**, *16*, 1989–1995, https://doi.org/10.1021/acs.nanolett.5b05263.
2. Grenadier, S.; Li, J.; Lin, J.; Jiang, H. Dry etching techniques for active devices based on hexagonal boron nitride epilayers. *J. Vac. Sci. Technol. A: Vacuum, Surfaces, Films* **2013**, *31*, 061517, https://doi.org/10.1116/1.4826363.
3. Wang, L.; Meric, I.; Huang, P.Y.; Gao, Q.; Gao, Y.; Tran, H.; Taniguchi, T.; Watanabe, K.; Campos, L.M.; Muller, D.A.; et al. One-Dimensional Electrical Contact to a Two-Dimensional Material. *Science* **2013**, *342*, 614–617.
